# The SPARK 2 Project: Developing a Clinical Academic Peer Group for Psychiatry Residents in the West Midlands Deanery

**DOI:** 10.1192/bjo.2026.11517

**Published:** 2026-06-30

**Authors:** Chantelle Wiseman, Shuqing Si, Ben Perry

**Affiliations:** University of Birmingham, Birmingham, United Kingdom

## Abstract

**Aims:**

To develop a peer group of clinical academic residents in the West Midlands Deanery.

The SPARK project (Supporting Psychiatric Academic Research for Knowledge) began in 2024 in the West Midlands Deanery to support resident doctors to achieve the research requirements of the higher training curriculum. From this work it became apparent that a smaller cohort of academicallyfocussed residents wanted support with applications and their additional training needs.

**Methods:**

We started a series of meetings for clinical academic residents and doctors interested in academic psychiatry careers working in the West Midlands. We held our first hybrid meeting in October 2025 at the University of Birmingham. This consisted of a hot networking lunch, a career talk from clinical Associate Prof Dr Ben Perry, a talk on theclinical academic training pathway and time for networking. We created a WhatsApp group where we share funding and job opportunities as they arise.

A second meeting is booked for March 2026 and will feature a career journey talk from Prof Femi Oyebode. This will be held at the University of Warwick so residents get exposed to different universities in the Midlands.

At the first meeting we measured pre-and post-session awareness of, and interest in, clinical academic careers.

**Results:**

21 participants recorded their attendance at the first SPARK 2 meeting, 16 in person and five online. The attendees were a mix of ACFs, core trainees, higher trainees, a foundation doctor, a clinical research fellow, an SAS doctor and a medical student.

Participants were asked to respond to the statement “I know what a clinical academic is and what they do”; 58% responded “Agree” or “Strongly agree” pre-session, 100% responded “Agree” or “Strongly agree” post-session. Participants were asked to respond to the statement “I understand the typical training and career pathway for a clinical academic”; 41% responded “Agree” or “Strongly agree” pre-session, 100% responded “Agree” or “Strongly agree” post-session. After the session 100% of participants responded “Agree” or “Strongly agree” with the statement “The seminar improved my understanding of clinical academia”.

One resident stated afterwards “That event really was pivotal” to his subsequent appointment as an ACF.

**Conclusion:**

SPARK 2 is a quality improvement project in the West Midlands to support clinical academic residents in the region. We run meetings and have created a WhatsAppgroup to allow for networking and the sharing of knowledge and resources.

